# Ionizing Radiation-Induced Extracellular Vesicle Release Promotes AKT-Associated Survival Response in SH-SY5Y Neuroblastoma Cells

**DOI:** 10.3390/cells10010107

**Published:** 2021-01-08

**Authors:** Flavia Tortolici, Simone Vumbaca, Bernadette Incocciati, Renu Dayal, Katia Aquilano, Anna Giovanetti, Stefano Rufini

**Affiliations:** 1 Department of Biology, University of Rome Tor Vergata, 00133 Rome, Italy; flavia.tortolici@hotmail.it (F.T.); simonevum41@gmail.com (S.V.); bincocciati@gmail.com (B.I.); katia.aquilano@uniroma2.it (K.A.); 2 Sanorva Biotech Private Limited, Mysuru 570008, India; renu.dayal10@gmail.com; 3 ENEA, Department of Energy and Sustainable Economic, 00123 Rome, Italy; anna.giovanetti@enea.it

**Keywords:** X-rays, exosomes, EVs, radiotherapy, radio-resistance, EMT, DNA damage, DNA repair, cancer, bystander effect

## Abstract

Radiation therapy is one of the most effective methods of tumor eradication; however, in some forms of neuroblastoma, radiation can increase the risk of secondary neoplasms, due to the ability of irradiated cells to transmit pro-survival signals to non-irradiated cells through vesicle secretion. The aims of this study were to characterize the vesicles released by the human neuroblastoma cell line SH-SY5Y following X-ray radiations and their ability to increase invasiveness in non-irradiated SH-SY5Y cells. We first purified the extracellular vesicles released by the SH-SY5Y cells following X-rays, and then determined their total amount, dimensions, membrane protein composition, and cellular uptake. We also examined the effects of these extracellular vesicles on viability, migration, and DNA damage in recipient SH-SY5Y cells. We found that exposure to X-rays increased the release of extracellular vesicles and altered their protein composition. These vesicles were readily uptaken by non-irradiated cells, inducing an increase in viability, migration, and radio-resistance. The same results were obtained in an *MYCN*-amplified SK-N-BE cell line. Our study demonstrates that vesicles released from irradiated neuroblastoma cells stimulate proliferation and invasiveness that correlate with the epithelial to mesenchymal transition in non-irradiated cells. Moreover, our results suggest that, at least in neuroblastomas, targeting the extracellular vesicles may represent a novel therapeutic approach to counteract the side effects associated with radiotherapy.

## 1. Introduction

Neuroblastoma is the second most common solid tumor in childhood, characterized by a highly aggressive behavior and poor prognosis. Due to the subtle and heterogeneous nature of its symptoms (e.g., weight loss, anemia, fever), most neuroblastomas have already metastasized at diagnosis to secondary sites such as the pelvis, neck, abdomen, or chest [1]. Therapeutic approaches include tumor resection, often combined with chemo and/or radiotherapy [2]. In case of radiation, the 21.6 Gy at 1.8 Gy per day, and the 21 Gy at 1.5 Gy per day represent the standard doses used in the clinic [2]. Despite slowing the course of the disease, the therapeutic use of ionizing radiations (IRs) has been frequently associated with an increased risk of developing secondary neoplasms [2].

In vivo and in vitro studies have shown that IRs can enhance the metastatic potential of many tumors by acting directly on cancer cells or on the tumor microenvironment, [3]. Sundahl et al. have recently described the molecular pathways by which IRs trigger the epithelial to mesenchymal transition (EMT), increase cancer cell dissemination, invasion, and adhesion, and ultimately promoting tumor aggressiveness [4]. How these experimental findings can be translated into the clinic is still not clear. However, in a few examples, such as non-small cell lung cancer, an increased number of circulating cancer cells was found in patients who have received radiotherapy [5].

In vitro studies have shown that cells not directly targeted by IRs accumulated genetic and epigenetic changes, which were mediated by gap junction or soluble factors released by the irradiated cells [6]. This process is well known as radio-induced bystander effect (RIBE). RIBE has also been observed in vivo, implying the need to revisit the current approaches to estimate radiation-associated health risks [7]. In bystander cells, RIBE has been shown to induce genomic instability, apoptosis, and senescence, as well as survival and adaptive response to many stresses, including IRs [8]. These indirect effects have been associated with the release of extracellular vesicles (EVs) by the irradiated cells [9].

EVs are a large family of elements defined by a double membrane containing proteins and nucleic acids of different types. The EV family comprises exosomes, apoptotic bodies, exosomes-like vesicles from mitochondria, and micro-vesicles deriving from the plasma membrane; as reviewed by Srivastava et al., the EV classification is constantly evolving [10]. Moreover, the amount and content of the EVs vary according to the cell state. Exosome-mediated communication between cancer cells has an active role in tumor progression [11]. Indeed, tumor-derived exosomes may increase cell migration and vascular permeability, shaping the metastatic niche and contributing to the survival of metastatic tumor cells [12]. Stress stimuli, including IRs, have been shown to increase the amount of EVs secreted by cells [13] and to modify their cargo [6], inducing a number of physiological and pathological processes and epigenetic changes [14]. In a recent review, Abramowicz et al. highlighted how stress conditions caused the release of EVs containing a protein cargo that was able to modulate critical processes in recipient cells, such as proliferation, survival, migration, and angiogenesis [15]. However, changes that are induced by IRs on EVs released by radio-resistant cells of the neuroblastoma lineage, and their impact on cancer evolution, have not been investigated so far.

The aims of this study were to characterize: (i) the amount, the protein composition, and uptake efficiency of EVs released by the human neuroblastoma cell line SH-SY5Y exposed to increasing doses of X-rays; (ii) the effects induced by these EVs on naïve SH-SY5Y, focusing on biological processes associated with metastasis, such as survival and migration. Finally, we compared the autocrine effects induced by EVs on cell proliferation and DNA repair in SH-SY5Y and the *MYCN*-amplified SK-N-BE neuroblastoma cell line.

In this study, we identify a key role of EVs released after X-ray exposure in activating cell survival pathways and inducing radio-resistance, which is associated with an increased capacity to repair DNA damage.

## 2. Materials and Methods

### 2.1. Cell Culture and Irradiation

The human neuroblastoma cell lines SH-SY5Y (ATCC^®^ CRL2266TM) and SK-N-BE (ATCC^®^ CRL-2271TM) were purchased from ATCC-LGC Standards (Middlesex, UK). Cells were cultured in Dulbecco’s Modified Eagle’s Medium (DMEM, Corning, Corning, NY, USA) supplemented with 10% fetal bovine serum (FBS, Corning), 2 mM l-glutamine (Sigma, St. Louis, MO, USA) and 1% (100 IU/mL penicillin and 100 μg/mL streptomycin) antibiotics (Sigma, St. Louis, MO, USA). Cell culture was maintained in a humidified atmosphere containing 5% CO_2_ at 37 °C. All experiments were carried out on undifferentiated neuroblastoma cells at a passage comprised between 10 and 15. Cells were rinsed with phosphate-buffered saline (PBS), dissociated with 0.05% trypsin and 0.02% EDTA, then plated according to the type of experiment. Cells were divided into different irradiation dose groups (0 Gy; 0.1 Gy; 1 Gy; 10 Gy). The day of the X-ray treatment, the standard culture medium was removed, and all cells, including controls (70–80% confluence) were washed once with PBS and then cultured in DMEM-containing exosome-depleted FBS (Gibco™, Thermo Fisher Scientific, Waltham, MA, USA). Then cells were irradiated with X-rays, with a CHF 320 X-rays generator (Gilardoni, Mandello del Lario LC, Italy), equipped with a 0.5 mm Cu filter, operating at 250 KeV and 15 mA, corresponding to 1 Gy/min, located in the laboratories of the ENEA-Casaccia Center (Rome, Italy).

### 2.2. Purification of EVs

Conditioned media from all the different experimental groups, including non-irradiated controls, were collected 3 h after irradiation. The day before irradiation, cells were seeded at a density of 5 × 10^4^ cells/cm^2^. At the time of supernatant collection, in each experimental point there were about 8 × 10^6^ cells. EVs were recovered from the supernatants by the filtration/ultracentrifugation protocol according to Théry et al. [16]. Medium collected from each experimental point was centrifuged at 3000× *g* for 30 min to remove floating cells and apoptotic bodies. Supernatants were filtered through 0.22 μm filters (Millipore) and ultra-centrifuged (L7-65 Ultracentrifuge, Beckman Coulter, Brea, CA, USA) using a SW28 rotor, at 100,000× *g* for 150 min at 4 °C. After the purification procedures, the EV pellet was resuspended in 200 μL of PBS and stored at −80 °C. The amount of EVs was estimated at 215 nm, corresponding to the absorbance peak of phospholipids, using known protein concentrations of commercial exosomes (HansaBioMed Life Science, Lona, Switzerland) as a standard curve [17]. All experiments were performed by pre-treating SH-SY5Y and SK-N-BE recipient cells with 0.04 μg/μL of EVs for 90 min and then irradiated/mock irradiated with X-rays (5 Gy). Each experiment was performed using a pool of independent EVs to execute independent biological triplicates.

All relevant data from our experiments were submitted to the EV-TRACK knowledgebase (EV-TRACK ID: EV200111) [18].

### 2.3. EVs Dimensional Characterization

A CytoFLEX, (Beckman Coulter, Brea, CA, USA) was used for EV characterization. Brittain et al. have shown that the CytoFLEX flow cytometer can efficiently detect nanoparticles and EVs from plasma samples highlighting the ability of this instrument to identify very small particles of 30–150 nm in diameter [19]. A standard Megamix-Plus SSC microparticles kit (BIOCYTEX, Marseille, France) was used for the EV detection. the Megamix-Plus kit contained nanosized FITC-A-conjugated standardized particles of different sizes: 100, 160, 200, 240, 300, 500, and 900 nm. Gates for data acquisition of all vesicle samples were set according to the manufacture’s instruction. The data were analyzed using Cytexpert 2.2 software (Beckman Coulter, Brea, CA, USA).

### 2.4. Immunoblotting

Cells were plated at the density of 2.5 × 10^4^ cell/cm^2^ in 6-well plates. The day of the experiment, after 24 h, cells were pre-treated for 90 min with EVs and subsequently irradiated with 5 Gy, or not. Thirty minutes or 24 h after X-ray treatment, cells were processed for viability and DNA damage repair assays, respectively. Cells and EVs were lysed in RIPA buffer (50 mM Tris–HCl, pH 8.0, 150 mM NaCl, 12 mM deoxycholic acid, 0.5% Nonidet P-40) containing a cocktail of protease and phosphatase inhibitors (Sigma, St. Louis, MO, USA). After protein quantification with the Lowry Method, sample buffer (final concentration: 50 mM Tris–HCl, pH 6.8, 2% SDS, 10% Glycerol, 0.02% Bromophenol blue, 1% β-mercaptoethanol) was added to the samples, which were boiled for 5 min at 90°. Next, 10 μg and 25 μg of proteins from the cells’ or EVs’ samples respectively, were loaded on SDS–PAGE under denaturing conditions (running condition 20 mA, 100 mV), and then subject to Western blotting analysis. Nitrocellulose membranes were incubated with anti-CD63 (EXOAB-CD63A-1, System Biosciences, Palo Alto, CA, USA), anti-FLOT1 (EXOAB-FLOT1-1, System Biosciences, Palo Alto, CA, USA), anti-CD9 (EXOAB-CD9-1, System Biosciences, Palo Alto, CA, USA), anti-CD81 (EXOAB-CD83A-1, System Biosciences, Palo Alto, CA, USA), anti-BAX (5023T, Cell Signaling Technology, Inc., Danvers, MA, USA), anti-ATM, anti-pATM, anti-BRCA1 (9947-DNA damage kit, Cell Signaling Technology, Inc., Danvers, MA, USA), anti-p53 (9282, Cell Signaling Technology, Inc., Danvers, MA, USA), anti-Tubulin (T9026, Sigma, St. Louis, MO, USA), anti-pPDK1 (C49H2, Cell Signaling Technology, Inc., Danvers, MA, USA), anti-AKT (9279, Cell Signaling Technology, Inc., Danvers, MA, USA), anti-pAKT (D25E6, Cell Signaling Technology, Inc., Danvers, MA, USA), anti-pFOXO1 (A27667, Cell Signaling Technology, Inc., Danvers, MA, USA), and anti-GAPDH (G9545, Sigma, St. Louis, MO, USA). All primary antibodies were used at 1:1000 dilution. Membranes were then incubated with the appropriate horseradish peroxidase-conjugated secondary antibodies at 1:500 dilution. Immuno-reactive bands were developed using the ECL Selected Western Blotting Detection Reagent (GE Healthcare, Pittsburgh, PA, USA) and detected by a FluorChem FC3 System (Protein-Simple, San Jose, CA, USA). Densitometric analyses of the immune-reactive bands were performed using the ImageJ Analysis Software version 1.50i (National Institutes of Health, Bethesda, MD, USA).

### 2.5. Cell Uptake of Labelled EVs

Purified EVs were labelled with Green SYTO^®^ RNASelectTM for RNA staining (Thermo Fisher Scientific, Waltham, MA, USA). Excessive unincorporated dye was removed from the labelled exosomes using Exosome Spin Columns (MW 3000) following the manufacturer’s protocol. For the fluorescence microscopy analysis, cells were cultured on coverslips in 12-well plates at a density of 1 × 10^5^ cells per well. After 24 h, SH-SY5Y cells were incubated with labelled/non-labelled EVs from 15 min to 2 h, then fixed with 2% PFA in PBS for 15 min at 4 °C. To visualize the cytoplasm, recipient cells were labelled with α-Tubulin antibody at 1:200 dilution (3873, Cell Signaling Technology, Inc., Danvers, MA, USA), incubated for 3 h at room temperature (RT) and then labelled with Goat anti-Mouse IgG Alexa Fluor^®^ 555 at 1:200 dilution. Finally, nuclei were stained for 10 min with 300 nM Hoechst 33342 (Thermo Fisher Scientific, Waltham, MA, USA). Specimens were imaged by a Nikon TE 2000 epifluorescence microscope equipped with a Photometrics CoolSNAP MYO CCD camera. For flow cytometry analysis, SH-SY5Y cells were cultured in 24-well plates (3 × 10^4^ cells/well), and after 24 h cells were incubated with labelled/non-labelled EVs from 15 min to 2 h, then harvested and washed with PBS. Cells were recorded in the FL-1 channel using a FACSCalibur flow cytometer (BD Biosciences, San Jose, CA, USA). The experiment was repeated three independent times, and the analysis was performed on 10,000 events using FlowJo, version 7.6.5 (FlowJo LLC, Ashland, OR, USA).

### 2.6. Cell Proliferation Assay

Cells were cultured in 96-well plates (5 × 10^3^ cells/well). Twenty four hours after treatments, 20 µL of the 3-(4,5-dimethylthiazol -2-yl)-5(3-carboxymethonyphenol)-2-(4-sulfophenyl)-2H tetrazolium (MTS) dye (Promega, Madison, WI, USA) was added to each well and incubated for 2 h at 37 °C in a humidified 5% CO_2_, 95% air mixture. Optical density (OD) was read directly at 490 nm using a Microplate Reader Tecan Sunrise (Tecan Group Ltd., Mannedorf, Switzerland). On the basis of preliminary data (Supplementary Data) the dose of EVs (0.04 µg/µL) was selected.

### 2.7. Cell Count

Cells were plated at a density of 2.5 × 10^4^ cells/cm^2^ in 24-well plates. After 24 h from the treatments, cells were detached and stained to evaluate cell viability by dye exclusion test, mixing 1 part of 0.4% trypan blue and 1 part cell suspension (dilution of cells).

### 2.8. Cell Cycle Analysis by Flow Cytometry

The neuroblastoma cells were seeded (2.5 × 10^4^ cells/cm^2^) and cultured in 24-well plates. After 24 h from the treatments, cells were harvested, washed with PBS and stained with 5 μg/mL of propidium iodide solution (Sigma, St. Louis, MO, USA) at 4 °C for 5 min in the dark. The DNA content was measured using a FACSCalibur flow cytometer (BD Biosciences, San Jose, CA, USA). The experiment was repeated three independent times, and the analysis was performed on 20,000 events using the MultiCycle DNA content and FlowJo (version 8.8.6.; FlowJo LLC, Ashland, OR, USA).

### 2.9. Cell Migration Assay

Migration of SH-SY5Y cells was carried out using the Ibidi Culture-Inserts assay (2 × 0.22 cm^2^; Ibidi, Regensburg, Germany) following the manufacturer’s instructions. Three hundred thousand cells were seeded on the culture-inserts. After 24 h, inserts were carefully removed and migration was analyzed using a BDS200 inverted microscope (Optec, Chongqing, China) at time 0 (insert removal) or following 24 and 48 h of EV treatment. To study the effects of AKT inhibition, cells were pre-treated with 10 μM of LY294002 (LY) (Cell Signaling Technology, Inc., Danvers, MA, USA) 1 h before EV treatment, and added every 24 h during medium refreshment. The migration rate was determined by analyzing the cell-free area with ImageJ Analysis Software 1.50i (National Institutes of Health, Bethesda, MD, USA).

### 2.10. RT-qPCR Analysis

SH-SY5Y cells were seeded and cultured in 24-well plates (2.5 × 10^4^). Twenty four hours after the treatments total RNA was extracted using TRI Reagent^®^ (Sigma, St. Louis, MO, USA) and 3 μg of RNA were retro-transcripted using M-MLV (Promega, Madison, WI, USA). RT-qPCR was performed in triplicate using Applied Biosystems^TM^ Power^TM^ SYBR^TM^ Green Master Mix and the QuantStudio3 Real-Time PCR System (Thermo Fisher, Whaltam, MA, USA). RT-qPCR analyses were performed with validated primers (BLAST) for Vimentin (forward: 5′-TCCCTGAACCTGAGGGAAAC-3′; reverse: 5′-AGGTCATCGTGATGCTGAGA-3′), N-Cadherin (forward: 5′-GCTGGACCGAGAGAGTTTCC-3′; reverse: 5′-CAAAATCCAAGCCCGTGGTG-3′) and mRNA levels were normalized to GAPDH (forward: 5′-AATGGGCAGCCGTTAGGAAA-3′; reverse: 5′-GCCCAATACGACCAAATCAGAG-3′). The relative mRNA levels were determined through the 2^−ΔΔ*C*t^ method.

### 2.11. Comet Assay

SH-SY5Y cells were seeded and cultured in 24-well plates (0.03 × 10^6^ cells/cm^2^). Alkaline comet assay is a single-cell gel electrophoresis method that allows the detection of both single and double strand DNA breaks [20]. Cells were pre-treated for 90 min with EVs and then X-ray irradiated. After 2 or 24 h, cells were resuspended in PBS containing 0.5% low melting point agarose, and pipetted onto a frosted glass microscope slide pre-coated with a layer of 0.2% normal melting point agarose. Slides were then incubated in an alkaline lysis solution (2.5 M NaCl, 10 mM Tris–HCl, 100 mM Na_2_EDTA, NaOH to pH 10, 1% Triton X-100, 10% DMSO) for 45 min; at this point, all the following steps were performed at 4 °C under dim light. After lysis, slides were rinsed for 10 min with electrophoresis buffer (1 mM Na_2_EDTA, 300 mM NaOH, pH 13),placed for 20 min onto a horizontal electrophoresis unit Sub-Cell GT System (15 × 25 cm) equipped with a Power Pack 300 (Bio-Rad Laboratories Inc., Hercules, CA, USA), and covered with 0.5 cm of electrophoresis buffer to allow DNA unwinding. Electrophoresis was carried out for 30 min (25 V, 300 mA). Subsequently, slides were gently rinsed in neutralization buffer solution for 5 min (0.4 M Tris–HCl, pH 7.5), dehydrated with an ethanol series (70%, 85%, and 100%), dried at RT, and stored. For microscopy analysis, slides were stained with ethidium bromide (10 µg/mL) immediately before being analyzed at 400× magnification using a fluorescent Axiolab Zeiss microscope (Carl Zeiss AG, Oberkochen, Germany).

For each experimental point, 500 cells were classified in 5 categories, according to the length of the comet tail that is proportional to the levels of DNA damage. DNA breaks were expressed as arbitrary units (AU) and reported in the text as fragmentation index (FI). Visual scoring is proportional to the amount of DNA present in the comet tail, as previously described [21].

### 2.12. Statistical Analysis

Each experiment was repeated at least three independent times. The results are presented as mean ± S.D. Statistical analyses were performed using the Student’s *t* test to compare the means of two groups. Differences were considered to be significant at *p* < 0.05. GraphPad software was used for the statistical analysis.

## 3. Results

### 3.1. Characterization of EVs

**Figure 1:**
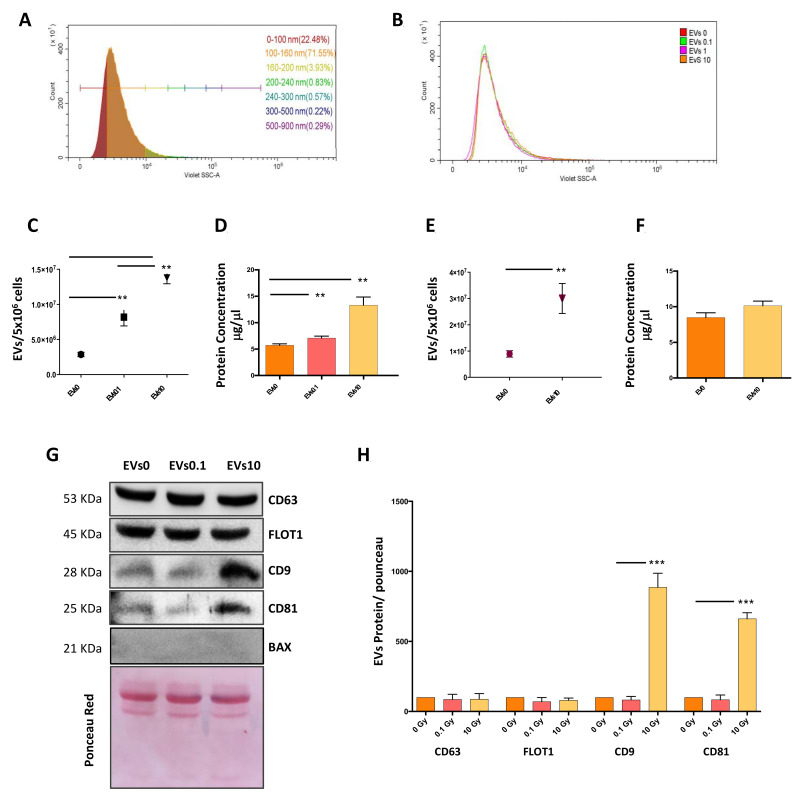
Characterization of extracellular vesicles (EVs) released by non-irradiated/irradiated neuroblastoma cells. (**A**) Representative histogram of the size distribution of EVs released by SH-SY5Y neuroblastoma cells. Size ranges are indicated by different colors in the horizontal bar; (**B**) Representative distribution dimension curves of EVs released by non-irradiated and irradiated neuroblastoma cells; (**C**) Quantitative analysis of SH-SY5Y EVs by flow cytometry (EVs/5 × 10^6^ cells); (**D**) Protein quantification of SH-SY5Y EVs; (**E**) Quantitative analysis of SK-N-BE EVs by flow cytometry (EVs/5 × 10^6^ cells); (**F**) Protein quantification of SK-N-BE EVs; (**G**) SH-SY5Y EVs were analyzed by Western blot to measure CD63, FLOT1, CD9, and CD81 protein levels. Ponceau Red staining and Bax were used as loading and negative control, respectively; (**H**) Densitometric relative to three western blots. Data are expressed as mean ± S.D. (n = 3, **, *p* < 0.01 ***, *p* < 0.001 vs. EVs0 or EVs0.1).

To evaluate whether IRs alter the number and morphology of EVs, we analyzed the EVs released by neuroblastoma cells after irradiation. To this end, we irradiated both SH-SY5Y and SK-N-BE cells with a range of X-ray doses, with the highest one (10 Gy) being comparable to what has been used in the clinic for the treatment of neuroblastoma [2]. Three hours after irradiation, media were collected and processed to isolate EVs. EVs from both neuroblastoma cell lines irradiated with X-rays at 0 (EVs0), 0.1 (EVs0.1), 1 (EVs1) and 10 Gy (EVs10), were monitored by a high-resolution flow cytometer using the side scatter of the violet laser (405 nm), which allows the detection of particles below 300 nm in diameter, corresponding to the size-range of exosomes. Analysis of EVs0, EVs0.1, EVs1, and EVs10 showed a similar size profile in both cell lines, characterized by a heterogeneous composition and an enrichment of particles with a diameter smaller than 160 nm (95%) (Figure 1A). Furthermore, all the dose groups showed overlapping size distribution curves (Figure 1B), indicating that the X-rays did not affect the average diameter of the EVs released by both neuroblastoma cell lines. Analysis of the number of events detected by flow cytometry revealed that the total amount of EVs directly correlated with the administered dose (*p* < 0.001 EVs10 vs. EVs0 and *p* < 0.01 EVs10 vs. EVs0.1 in SH-SY5Y cells, and *p* < 0.01 EVs10 vs. EVs0 in SK-N-BE cells) (Figure 1C–E). Accordingly, the EVs protein concentration increased with the X-ray doses. This increase was significant in SH-SY5Y cells, while a similar trend was observed in SK-N-BE cells (Figure 1D–F). Western blot analysis of specific EV protein markers (CD63, FLOT1, CD9, and CD81) revealed that CD63 and FLOT1 protein levels were similar in all the EVs isolated from SH-SY5Y cells (Figure 1G,H). In contrast, the tetraspanins CD9 and CD81 protein levels were significantly higher in the EVs10 compared to the other groups (Figure 1G,H). Similar results were obtained in the SK-N-BE cells (data not shown), confirming that the two cell lines produced similar EVs when irradiated with X-rays. These experiments showed that X-rays increased the number of released EVs, as well as the exosome fraction.

### 3.2. EVs Released by Irradiated Cells Are Efficiently Up Taken

**Figure 2:**
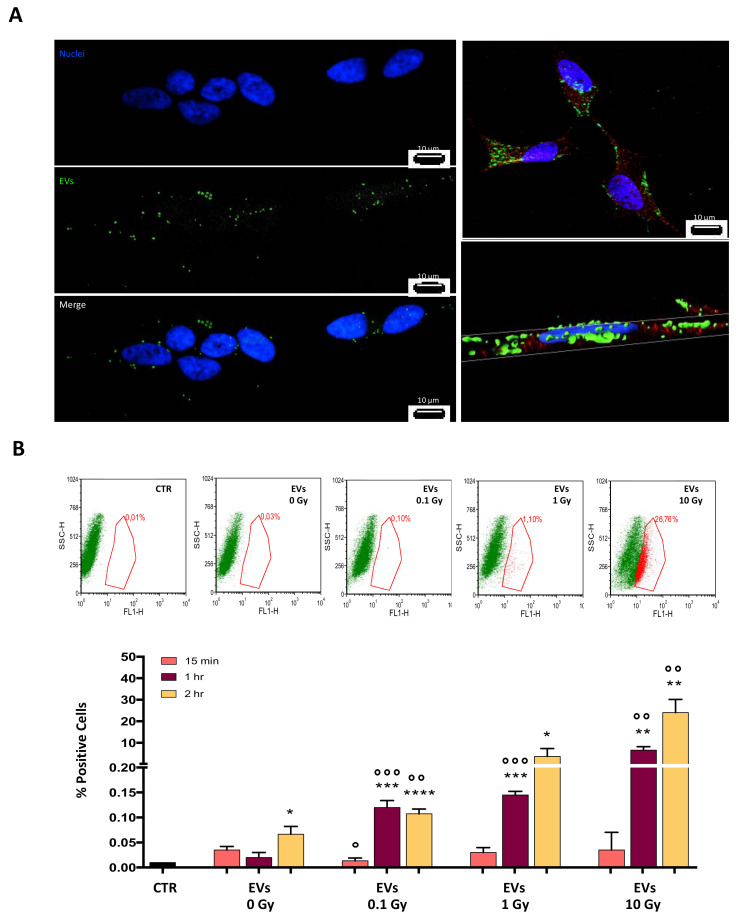
EV uptake by recipient cells. (**A**) SH-SY5Y were incubated with Green SYTO^®^ RNASelectTM-labelled EVs, and imaged by fluorescence and confocal microscopy (left and right panel respectively); cytoplasm and nuclei were stained with α-Tubulin and blue Hoechst 33342, respectively. The right panel represents the three-dimensional reconstructed images of the confocal microscopy analysis; (**B**) SH-SY5Y were incubated with Green SYTO^®^ RNASelectTM-labelled EVs and analyzed by flow cytometry. Representative FL1-H vs. SSC-H dot plots showing the gating strategy (red boxes) used to determine cells that had incorporated the labelled EVs (upper panel). Flow cytometry analysis of labelled EVs uptake by SH-SY5Y cells after 15 min, 1 h, and 2 h treatment (bottom panel). Data are expressed as means ± S.D. (n = 4, * *p* < 0.05, ** *p* < 0.01, *** *p* < 0.001, **** *p* < 0.0001 vs. 15 min uptake; n = 4, ° *p* <0.05, °° *p* < 0.01, °°° *p* < 0.001 vs. 0 Gy).

To evaluate the EV uptake by recipient cells, we performed a confocal microscopy analysis of SH-SY5Y cells incubated with fluorescent-labelled EVs. Two hours after incubation, the fluorescent-labelled EVs were incorporated into the recipient cells as shown by the fluorescence images (Figure 2A, left panel).

A three-dimensional reconstruction of the image (Figure 2A, right panel) showed that the green granules were inside the cytoplasm, identified by the α-Tubulin staining (red) and in the same plane as the nucleus, highlighted by the Hoechst 33342 dye (blue), indicating their localization within the cell. It should be noted that the green fluorescence appeared as granules, suggesting that EVs were assembled in particulate structures throughout the cytoplasm.

Quantitative analysis of the incorporation kinetics of labelled EVs was carried out by flow cytometry at 15 min, 1 h, and 2 h after addition of EVs to SH-SY5Y cells (Figure 2B). EV uptake increased with the incubation time, and the dose delivered to the donor cells; after 2 h, EVs 0.1 and EVs10 showed an incorporation of about 12-times, and at least 200-times, higher than EVs0, respectively.

### 3.3. EVs Increase Cell Viability of Recipient Cells

**Figure 3:**
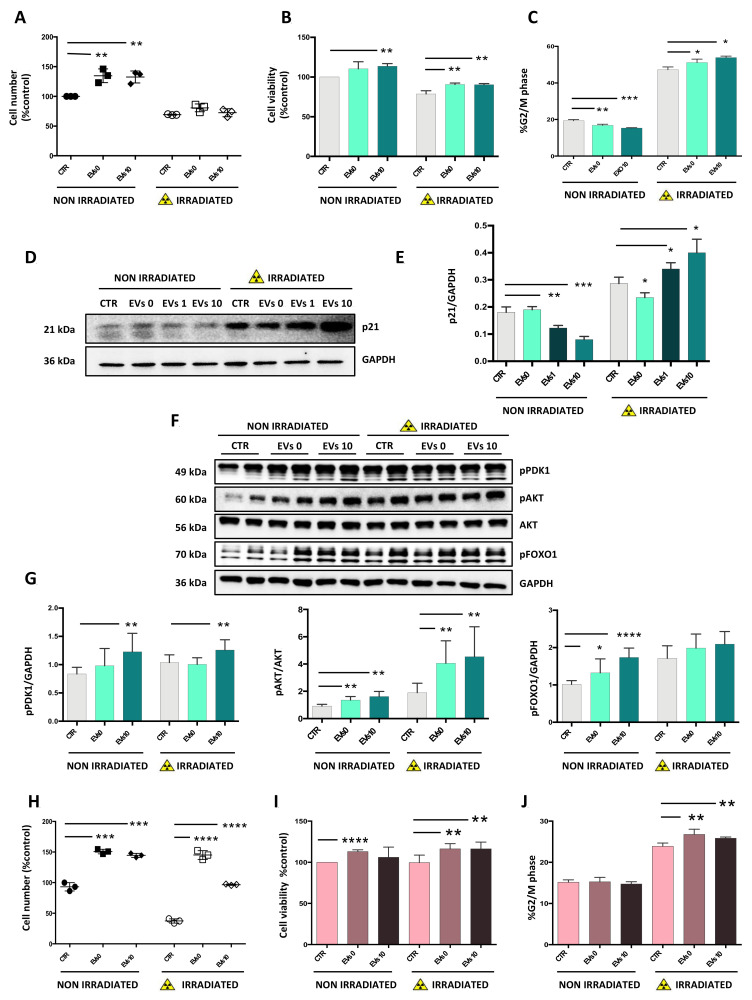
Effects of EVs on viability of recipient cells. Neuroblastoma cells were pre-treated for 1 h with EVs0 or EVs10, and then exposed/mock exposed to X-rays. (**A**) Cell count of SH-SY5Y cells was determined by Trypan blue exclusion assay. (**B**) Viability of SH-SY5Y cells was assessed by the MTT assay 24 h after irradiation. Absorbance was measured at 490 nm. (**C**) Percentages of SH-SY5Y cells in G2-M phase was determined by flow cytometry analysis; (**D**) Protein levels of p21 in SH-SY5Y cells, and (**E**) densitometric relative to three western blots. (**F**) Protein levels of pPDK1, pAKT (T308), AKT, pFOXO1 (Ser256) in SH-SY5Y cells, and (**G**) densitometric relative to three western blots. (**H**) Cell count of SK-N-BE cells determined by Trypan blue exclusion assay. (**I**) Viability of SK-N-BE cells measured by the MTT assay 24 h after irradiation. Absorbance was measured at 490 nm; (**J**) Percentages of SK-N-BE cells in G2-M phase determined by flow cytometry. Data are expressed as means ± S.D. (n = 4, * *p* < 0.05, ** *p* < 0.01, *** *p* < 0.001, **** *p* < 0.0001 vs. CTR).

The effect on viability induced by EVs was assessed in SH-SY5Y cells that were irradiated/non-irradiated with 5 Gy of X-rays. Trypan blue exclusion assay showed that in non-irradiated SH-SY5Y cells, 24 h exposure to EVs induced about a 20% increase in the number of viable cells compared to untreated cells, while no difference was observed in irradiated SH-SY5Y cells (Figure 3).

Viability was measured by the reduction of tetrazolium dye to formazane (MTT test). The MTT assay showed a significant increase in viability in non-irradiated SH-SY5Y cells treated with EVs10 compared to untreated cells. Moreover, in SH-SY5Y cells exposed to 5 Gy of X-rays, the viability of cells treated with EVs0 and EVs10 was significantly higher than untreated cells. We also performed cell cycle analysis by flow cytometry in irradiated/non-irradiated SH-SY5Y cells exposed to EVs. The number of cells in the G2-M phase of the cell cycle was significantly lower in samples incubated for 24 h, with both EVs0 and EVs10. Moreover, 5 Gy of X-rays induced about a 2.5-fold increase in the G2-M cell population and incubation with EVs further increased the number of cells in G2-M compared to control (Figure 3C). We then carried out a Western blot analysis of p21, a known mediator of cell cycle arrest in G2-M. In non-irradiated cells incubated with EVs1, and even more in those treated with EVs10, the levels of p21 protein significantly decreased compared to control. After 5 Gy of X-rays, p21 levels increased, and this increase was more pronounced in EVs1 and EVs10 treated-cells (Figure 3D,E).

The early transduction events triggered by EVs were examined by analyzing the phosphorylation levels of AKT [T308] and FoxO1 [S256]. In non-irradiated cells, a rapid phosphorylation of AKT, mediated by PDK1, was observed after 3 h incubation with EVs0 and EVs10 (Figure 3F,G). Following X-rays, the levels of AKT phosphorylation in EVs0 and EVs10 treated-cells was significantly higher when compared to control and to non-irradiated cells.

The autocrine effect of EVs on cell proliferation was then analyzed in SK-N-BE cells. Cell count (Figure 3H), MTT assay (Figure 3I), and cell cycle analysis (Figure 3J), yielded results similar to those observed in SH-SY5Y cells. However, there was a considerable difference in the effects of EVs on the radio-induced cell death. While in SH-SY5Y cells, pre-treatment with EVs0 and EVs10 did not change the post-radiation number of viable cells, in SK-N-BE both EVs0 and EVs10 appeared to induce a substantial radio-resistance (Figure 3H—right side). All together, these results showed a similar behavior in the two cell lines, and confirmed the role of EVs in stimulating proliferation and cell survival.

### 3.4. EVs from Irradiated Cells Increase Cell Migration

**Figure 4:**
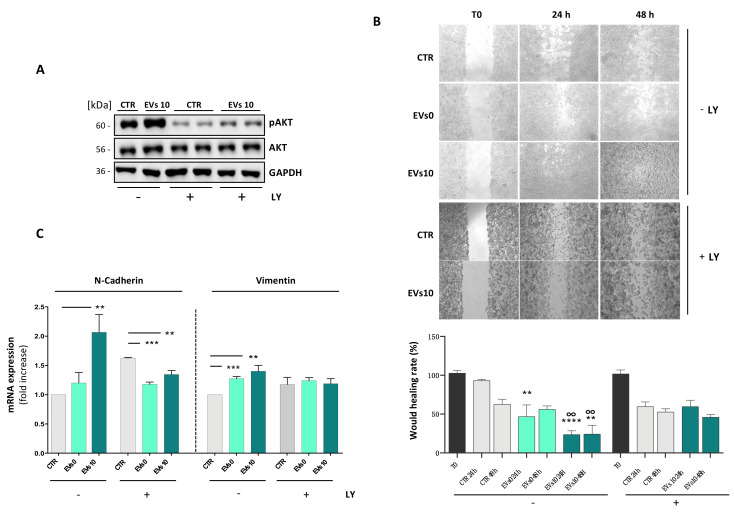
AKT activation is required for EVs-induced SH-SY5Y migration. SH-SY5Y cells were treated/mock treated with LY and then exposed to EVs0 or EVs10. (**A**) Protein levels of pAKT (T308) and AKT were determined by Western blotting using GAPDH as loading control. (**B**) Wound-healing assays were performed using Ibidi Culture-Inserts. Cell migration was recorded at 24 and 48 h after the inserts were removed (upper panel); wound healing rate (bottom panel). (**C**) mRNA expression of N-Cadherin and Vimentin was determined by RT-qPCR. Data are expressed as means ± S.D. (n = 3, ** *p* < 0.01, *** *p* < 0.001, **** *p* < 0.0001, vs. CTR; n = 3, °° *p* < 0.01, vs. EVs0).

To assess the effects of PI3K inhibition on EMT, cells were pre-treated for 1 h with 10 μM LY and then exposed to EVs. A wound-healing assay was used to investigate the effects of EVs on cell migration. In SH-SY5Y cells, incubation with 10 μM LY greatly reduced AKT phosphorylation [T308] levels in both control and cells grown in the presence of EVs (Figure 4A). To study the ability of cells to close the gap, SH-SY5Y cells were grown for 24 and 48 h in exosome-free medium or in medium containing EVs0 or EVs10. As shown in Figure 4B, SH-SY5Y cells grown in exosome-free medium showed a lower migration rate than cells grown in medium containing EVs. Moreover, cells treated with EVs10 migrated faster than those treated with EVs0. Pre-incubation of cells with the AKT inhibitor, LY, hindered the stimulatory effect of EVs on cell migration (Figure 4B). Vimentin and N-Cadherin are two important EMT markers that regulate cell migration. Figure 4C shows that cells treated with EVs expressed a significantly greater amount of Vimentin and N-Cadherin mRNAs compared to non-treated cells. This result is in line with the observations that cells stimulated with EVs have a greater migratory capacity than non-stimulated cells.

### 3.5. EVs from Irradiated Cells Increase the Rate of DNA Break Repair

In order to investigate the role of EVs in protecting cells from radio-induced DNA damage, we evaluated DNA breaks and repair by Comet Assay. SH-SY5Y cells were incubated for 1 h without EVs or with EVs0, EVs0.1, EVs1, or EVs10, and then irradiated with 5 or 10 Gy of X-rays.

**Figure 5:**
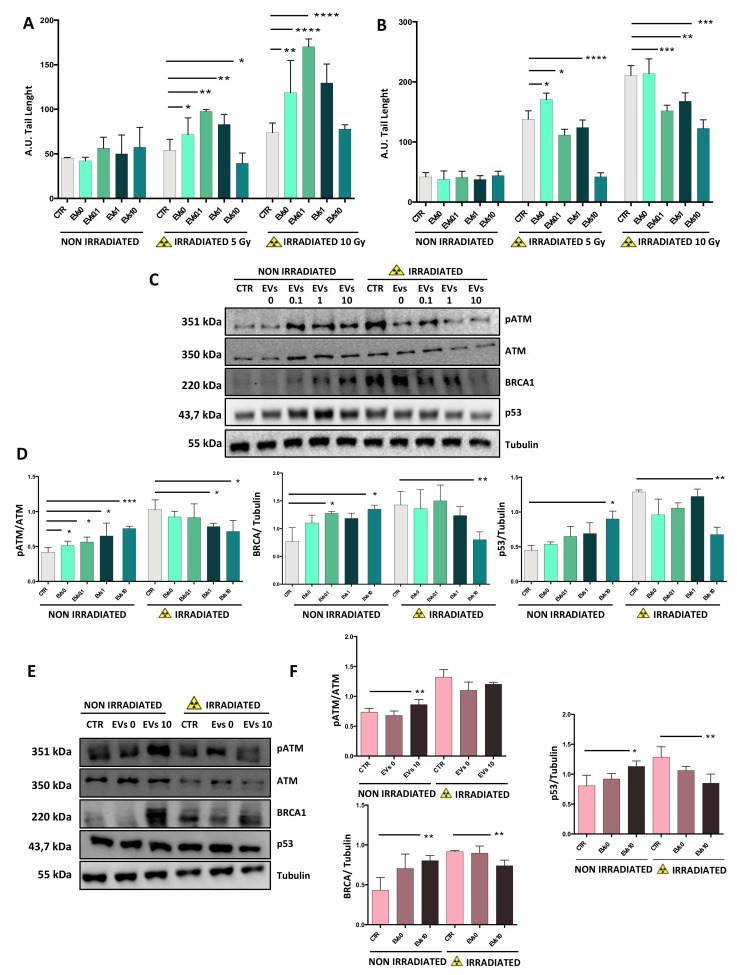
EVs protect cells from radio-induced DNA damage. Neuroblastoma cells were pre-treated for 1 h with EVs0, EVs0.1, EVs1, and EVs10, and then exposed/mock exposed to X-rays. SH-SY5Y cells were processed for Comet Assay after 2 h (**A**) and 24 h (**B**); (**C**) pATM (S1981), ATM, BRCA1, and p53 protein levels in SH-SY5Y cells were determined by Western blotting using Tubulin as loading control. (**D**) Relative densitometric analysis of the above-mentioned proteins. (**E**) pATM (S1981), ATM, BRCA1, and p53 protein levels in SK-N-BE cells were determined by Western blotting using Tubulin as loading control. (**F**) Relative densitometric analysis of the above-mentioned proteins. Data are expressed as means ± S.D. (n = 4, * *p* < 0.05, ** *p* < 0.01, *** *p* < 0.001, **** *p* < 0.0001 vs. 6 CTR).

DNA damage was analyzed after 2 and 24 h. All non-irradiated cells showed very low levels of DNA damage (Figure 5A). After 2 h, control cells showed an increase in DNA damage only after 10 Gy, while in EVs0, EVs1, and to a major extent in EVs0.1 treated-cells, DNA damage increased with the X-ray dose. In EVs10-treated cells, the radio-induced DNA damage was significantly lower, and increased only after exposure to 10 Gy, with a number of DNA breaks similar to control cells. DNA damage in control and EVs0-treated cells was much higher at 24 h compared to 2 h after irradiation, increasing significantly with the irradiation dose (Figure 5B).

In EVs10 treated-cells, DNA damage detected at 24 h after irradiation was significantly lower compared to all the other EV groups, and increased only after exposure to 10 Gy. Key DNA repair proteins (pATM, BRCA1, and p53) were analyzed in non-irradiated cells and 24 h after 5 Gy X-ray exposure (Figure 5C,D). These recipient cells were treated/mock treated with EVs0, EVs0.1, Evs1, or Evs10. In non-irradiated recipient cells, EVs induced an increase in the expression levels of DNA repair proteins that was directly proportional to the irradiation dose given to the donor cells. In contrast, an inverse trend was observed in irradiated recipient cells, where the EVs induced a decrease in the expression levels of DNA repair proteins that was inversely proportional to the irradiation dose given to the donor cell.

Similar observations were obtained in SK-N-BE cells, suggesting that the presence of the *MYCN*-amplification does not modify the cellular response to EVs, or even to X-rays (Figure 5E,F).

## 4. Discussion

The aim of this study was to examine whether exposure to IRs stimulates a dose dependent release of EVs, containing an increased exosomes’ fraction in SH-SY5Y neuroblastoma cells, and to analyze the biological effects induced by these EVs. We also investigated the role played by MYC, by comparing some of the main results with those obtained on the MYCN-amplified SK-N-BE neurobolastoma cell line.

Regarding the first point, we found that EVs released from irradiated cells expressed different amounts of membrane protein markers; indeed, while FLOT1 and CD63 were present in all experimental groups, CD9 and CD81 were mainly detected in the EVs from irradiated cells. Using a proteomic and flow cytometric approach, CD63, FLOT1, CD9, and CD81 were shown to be specific of the vesicular secretome of SH-SY5Y cells [22]. However, while FLOT1 and CD63 were present in the whole extracellular vesicular subtype, CD9 and CD81 were found preferentially in the exosome fraction [23]. Different protein composition of the exosomes released by other tumor or normal cells after irradiation, has also been described by Yentrapalli et al. [24] and Abramowicz et al. [25]. Interestingly, CD81 and CD9, whose expression increases in EVs10 compared to EVs0, were both related to the modulation of cell migration linked to PI3K activation [26,27]. The differences in the membrane protein composition that we observed, may reflect the previously described changes in the potential surface of EVs released by SH-SY5Y cells after irradiation [28].

The EVs diameter, measured by CytoFLEX, did not show any significant difference in size distribution between the different dose groups, in agreement with our previous observations using DSL [28]. More than 80% of EVs were in the range of 40–160 nm, which, according to the ISEV guidelines, represents the size of the exosomes [29]. These results are in agreement with studies in other cell lines showing no differences in EVs size distribution after exposure to IRs [30,31]. Following IRs, SH-SY5Y cells released a higher amount of EVs, which was directly related to the administered dose. An increase in exosome release following various stresses, such as physical agents (IRs), chemical drugs, and contaminants, has been reported in many cell lines [32]. This increase was associated with higher expression levels of p53 protein, which acts as an activator of the exosomes biogenesis [33]. Likewise, the increase in p53 expression levels that we observed in irradiated SH-SY5Y cells may be linked to the higher number of exosomes released by these cells. Altogether, our results show that after irradiation, the EVs fraction released by the SH-SY5Y cells is enriched in exosomes. We also found that EVs were quickly up taken by the SH-SY5Y cells. Preliminary observations using confocal microscopy indicated that the EVs uptake follows a complex kinetic: by 3 h of incubation, the internalized fluorescent probes appeared to cluster in granules; afterwards, the fluorescence intensity progressively decreased, and was detected as a diffuse signal throughout the whole intracellular space (data not shown). We therefore analyzed the EVs uptake by flow cytometry after 3 h of incubation. The analysis shows that EVs released by irradiated SH-SY5Y cells were incorporated more efficiently than the EVs released by non-irradiated cells. This difference had already been reported in other tumor cell lines [34]. The reason underlying this efficient uptake may be related to specific biochemical modifications of the membranes in the EVs released by irradiated cells. This topic, which goes beyond the interests of our research, is of fundamental importance for the use of EVs as carriers of pharmacologically active molecules [35]. An integrated proteomic and cell biology approach is needed to foster our knowledge on the use of EVs for therapeutic purposes.

Our second aim was to investigate the effects of the EVs secreted by irradiated SH-SY5Y cells on proliferation, migration, and response to IRs. An autocrine EVs stimulating effect has been already reported in other tumor cell lines and in cancer patients [36,37]. We found that in SH-SY5Y cells, EVs10 stimulated proliferation, in line with what has been reported in different cell lines [34], although this effect has not been observed in all tumor cells [38,39,40]. The discrepancy of these results may be due to several factors, including the type of cancer cells used, the radiation dose administered, and the method employed to purify the EVs. An important element to consider is the time elapsed between irradiation and EVs isolation. In our study, EVs were purified from the cell medium shortly (3 h) after irradiation, thus reflecting the early response of SH-SY5Y to the IR challenge.

Data obtained from the MTT, flow cytometry, and cell count analyses indicate that EVs have a stimulatory effect on cell viability and proliferation. Three hours after EVs treatment, we observed a decrease in the amount of p21 protein levels and an increase in FOXO1 phosphorylation, in agreement with the stimulation of cell cycle progression [41]. These EVs-mediated effects are likely downstream of the PI3K pathway, as shown by the increase in PDK and AKT phosphorylation. In irradiated cells that were pre-incubated with EVs, a complex cross-talk takes place between the IR- and EVs-dependent pathways. In particular, we observed a synergistic increase in AKT phosphorylation and a restoration of both p21 and FOXO1 protein levels. The final outcome is a significant accumulation of cells in the G2-M phase of the cell cycle, which represents a typical post-IR response for enabling the DNA repair pathway.

We identify the EVs as the autocrine mechanism that enhances and supports SH-SY5Y neuroblastoma cell migration. Moreover, this stimulatory effect is more evident in cells treated with EVs10. Not only is cell migration fundamental to many physiological processes, such as immune response and wound healing, but its activation in cancer promotes metastases [42,43]. Our data strongly suggest that the EVs10-induced migration is the result of AKT activation. Indeed, inhibition of AKT phosphorylation by LY impairs the ability of EVs10 to stimulate motility of SH-SY5Y cells. In support of this hypothesis, we observed that the mRNAs encoding for Vimentin and N-Cadherin, two proteins associated with migration, were increased by the EVs10 treatment, and that this increase was blocked following inhibition of AKT phosphorylation. Therefore, our observations are in agreement with several studies reporting that AKT plays a central role in cancer metastases [44].

EVs released by both irradiated and non-irradiated SH-SY5Y cells do not change the basal levels of DNA damage in recipient cells. This finding is in contrast with other studies showing that exosomes from irradiated cells are able to promote RIBE both in vivo and in vitro [6,14,45]. Recently, Ariyoshi et al. demonstrated that RIBE is mediated by exosome-like vesicles released by irradiated cells and, in particular, by their load of mutated mtDNA [45]. Consistent with these results, cells depleted of mitochondria do not produce RIBE signals [46]. The absence of RIBE that we observed in SH-SY5Y cells may be related to their strong anaerobic metabolism and their limited mitochondria content [47]. When the EV-treated SH-SY5Y cells were exposed to 5 or 10 Gy of X-rays, the amount of DNA breaks changed depending on the X-ray dose administered to the donor cells. Indeed, 2 h after irradiation, SH-SY5Y cells incubated with EVs0, EVs0.1, and EVs1 showed higher DNA damage compared to cells treated with exosome-depleted medium or EV10. We assume that the amount of DNA breaks observed at 2 h post IRs, which represents the fastest technical time to perform the Comet assay, reflects the early damage induced by IRs or, more likely, by radio-induced oxygen radicals. For this reason, we speculate that EVs deriving from low-dose irradiated cells interfere with IRs-induced oxygen radical production, either directly or through mitochondrial impairment. However, when we analyzed DNA damage 24 h after irradiation, when the DNA repair mechanisms had already been activated, we observed that in cells treated without EVs or with EVs0, DNA damage was still high. In contrast, in cells treated with EVs10, and to a lesser but significant extent with EVs1 and EVs0.1, DNA damage is largely repaired. This trend mirrors the expression levels of some of the key proteins involved in the DNA repair pathways. Indeed, pATM, BRCA1, and p53 are over-expressed in cells treated with EVs (increasing from EVs0.1 to EVs10) compared to those treated with exosomes-depleted medium before irradiation, suggesting a dose dependent induction of the DNA repair machinery. Twenty four hours after irradiation, when most of the DNA damage in EV-treated cells was repaired, the levels of the DNA repair proteins were higher in untreated cells, where DNA damage was not still completely repaired. Exosome-induced radio-resistance has also been observed in other tumor cell lines [15]. Moreover, a role of exosomes, released from irradiated cells, in enhancing DNA repair has been proposed in head and neck cancer [34].

Amplification of the *MYCN* is a critical prognostic factor in neuroblastoma, while high expression levels of N-Myc protein have been involved in resistance to anticancer treatments. Notably, almost 30% of neuroblastoma patients show the *MYCN* amplification [48]. The correlation between oncogene amplification and increased tumor aggressiveness has been extensively studied; nevertheless, since *MYC* is involved in several cellular pathways, the role of this oncogene in tumor progression has not yet been fully elucidated [49,50]. Accordingly, in this study we found that the autocrine response of *MYCN*-amplified SK-N-BE cells to EVs was similar or even greater than the one observed in SH-SY5Y cells. This enhanced response may be explained by the higher number of EVs we found to be released by the SK-N-BE cells, which are capable of stimulating survival, and inducing radio-resistance in recipient cells.

Recently, Aravindan et al. have published a comprehensive review on the key role of microRNAs-loaded exosomes in neuroblastoma multi-drug resistance [50]; other authors have investigated pathways in neuroblastoma cells that are important in determining radio-resistance [51]. Abramowicz et al. reviewed the role of EVs released by cells subjected to environmental physicochemical stress in regulating proliferation, cell survival, migration, and angiogenesis [15]. However, to our knowledge, this work together with Cerreto et al. [28] are the first studies proving that EVs released shortly after X-rays radiation can induce the activation of survival pathways that correlate with EMT in recipient cells.

## 5. Conclusions

In conclusion, here we demonstrated that EVs released early after irradiation by neuroblastoma cells can induce the activation of survival pathways that increase proliferation and invasiveness associated with the EMT in recipient cells. When these cells are irradiated, EVs induce an accumulation of cells in the G2/M phase of the cell cycle, allowing the DNA to be repaired. Our results are in agreement with many studies reporting an IRs-dependent increase in AKT phosphorylation levels, suggesting an adaptive cell response that, along with the other outcomes, leads to a more aggressive and radio-resistant phenotype. Radiation therapy is one of the most effective methods of tumor eradication, but it can also have deleterious effects. Therefore, our data point to EVs and/or their downstream dependent survival pathways as possible targets to counteract radio-resistance.

## Data Availability

All the data that support the findings of this study are from the corresponding author upon reasonable request.

## Supplementary Materials

The following are available online at https://www.mdpi.com/2073-4409/10/1/107/s1.
