# Supplementary material for: Ionizing Radiation-Induced Extracellular Vesicle Release Promotes AKT-Associated Survival Response in SH-SY5Y Neuroblastoma Cells
Source: Cells. 2021 Jan 8;10(1):107. doi: 10.3390/cells10010107 (PMC7827279; doi:10.3390/cells10010107)
Supplement: Supplementary file 1 [file cells-10-00107-s001.pdf]

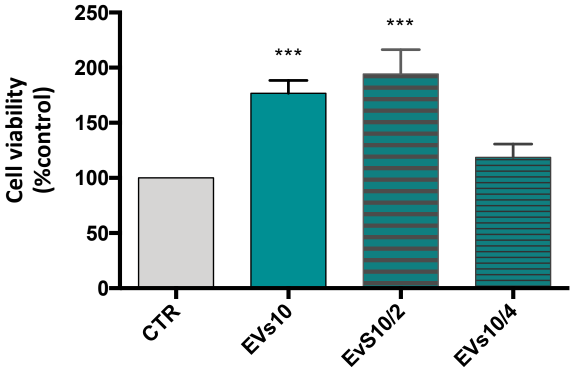

Viability measured incubating cells with different concentration of EVs10.

Cells were cultured in 96-well plates (5×103 cells/well). After 24 h cells, excepting controls, were treated with EVs10 at different concentration: 0.04 µg/µl (EVs10), with 0.02 µg/µl (EVs10/2), and with 0.01 µg/µl (EVs10/4).

After 24 h, 20 µl of MTS dye (Promega, Madison, WI, USA) was added to each well and incubated for 2 h at 37°C, in a humidified 5% CO2, 95% air mixture. Optical density (OD) was read directly at 490 nm using the Microplate Reader Tecan Sunrise (Tecan Group Ltd, Switzerland). Data are expressed as means ± S.D. (n=4, ***p<0.001 vs CTR).
